# Atopic Dermatitis-like Genodermatosis: Disease Diagnosis and Management

**DOI:** 10.3390/diagnostics12092177

**Published:** 2022-09-09

**Authors:** Chaolan Pan, Anqi Zhao, Ming Li

**Affiliations:** 1 Department of Dermatology, Xinhua Hospital, Shanghai Jiaotong University School of Medicine, Shanghai 200092, China; 2 Institute of Dermatology, Shanghai Jiaotong University School of Medicine, Shanghai 200092, China; 3 Department of Dermatology, The Children’s Hospital of Fudan University, Shanghai 200092, China

**Keywords:** atopic dermatitis, differential diagnosis, genetic diseases

## Abstract

Eczema is a classical characteristic not only in atopic dermatitis but also in various genodermatosis. Patients suffering from primary immunodeficiency diseases such as hyper-immunoglobulin E syndromes, Wiskott-Aldrich syndrome, immune dysregulation, polyendocrinopathy, enteropathy, X-linked syndrome, STAT5B deficiency, Omenn syndrome, atypical complete DiGeorge syndrome; metabolic disorders such as acrodermatitis enteropathy, multiple carboxylase deficiency, prolidase deficiency; and other rare syndromes like severe dermatitis, multiple allergies and metabolic wasting syndrome, Netherton syndrome, and peeling skin syndrome frequently perform with eczema-like lesions. These genodermatosis may be misguided in the context of eczematous phenotype. Misdiagnosis of severe disorders unavoidably affects appropriate treatment and leads to irreversible outcomes for patients, which underlines the importance of molecular diagnosis and genetic analysis. Here we conclude clinical manifestations, molecular mechanism, diagnosis and management of several eczema-related genodermatosis and provide accessible advice to physicians.

## 1. Introduction

Atopic dermatitis is one of the most prevalent inflammatory skin diseases, affecting 15% to 20% among children and up to 10% among adults [1]. The pathogenesis of atopic dermatitis is complex, involving interactions among genetic and environmental factors, epidermal barrier dysfunction, immune dysregulation, and microbial imbalance [2]. Atopic dermatitis is characterized by chronic, recurrent, and pruritic eczema, often with seasonal fluctuations. The skin lesions can manifest as erythema, papules, oedema, crusting, scaling, and hyperpigmentation and/or hypopigmentation. Associated clinical signs show dry skin, ichthyosis, lichenification and hyperlinear palms. Other manifestations of allergies such as food allergy, asthma and allergic rhinoconjunctivitis may occur. Atopic dermatitis is a clinical diagnosis, and its renewed diagnosis criteria formulated by the American Academy of Dermatology consist of three sections: (1) essential features that must be present for diagnosis including chronic or relapsing history, eczema (acute, subacute, chronic), pruritus, and typical morphology and age-specific patterns of skin lesions; (2) important features that support the diagnosis, such as atopy (personal or family history), early-onset age, IgE reactivity, and xerosis; and (3) associated features that is suggestive for the diagnosis but nonspecific, such as hyperlinear palms, lichenification. Indeed, atopic dermatitis-like lesions are frequently present in several genoderamtosis associated with immunological, metabolic or keratinization dysfunctions. In this review, the genetic disorders with eczematous phenotype are divided into three categories, including immunodeficiency related diseases, inherited metabolic diseases, and rare syndromes. We discuss the genetic molecular mechanisms and clinical manifestations of these diseases, and further provide feasible advice for their differential diagnosis and managements.

## 2. Immunodeficiency Related Diseases

Primary immunodeficiency diseases (PIDDs) is a group of mostly monogenic disorders with immune system dysfunction, characteristically presenting with recurrent infections [3]. As for skin manifestations, atopic dermatitis-like lesion is a common finding among several PIDDs, being reported in 13% to 57% of patients with PIDDs in previous studies [4,5,6,7]. In particular, the skin lesion might be the early or heralding manifestation of some immunodeficiency diseases, emphasizing the importance to distinguish it from atopic dermatitis and detect the underlying life-threatening immunologic defects [4]. Here, we focus on the PIDDs commonly presented with atopic dermatitis-like skin lesions, including hyper-immunoglobulin E (IgE) syndromes (HIES), Wiskott-Aldrich syndrome (WAS), immune dysregulation, polyendocrinopathy, enteropathy, X-linked (IPEX) syndrome, STAT5B deficiency, Omenn syndrome (OS), atypical complete DiGeorge syndrome, and X-linked agammaglobulinemia (XLA).

### 2.1. Hyper-IgE Syndromes

Characterized by a triad of eczema, recurrent infections along with elevated IgE levels, hyper-IgE syndromes refer to a heterogeneous group of monogenic inborn immune disorders with either autosomal dominant (AD) or autosomal recessive (AR) inheritance, namely AD-HIES or AR-HIES, respectively [8,9]. Great progress in the identification of pathogenic genes has been made over the past decade. Heterozygous mutations in the *signal transducer and activator of transcription-3* (*STAT3*) gene with dominant negative (DN) effect are recognized as the classical AD-HIES [10,11], also known as STAT3-HIES or Job syndrome. Biallelic mutations in the *phosphoglucomutase 3* (*PGM3*) [12] and the *dedicator of cytokinesis-8* (*DOCK8*) [13,14] are common causes of AR-HIES. In the 2019 update of the International Union of Immunological Societies (IUIS) genetic and phenotypical classification [15], five newly identified monogenic disorders with mutations associated with the STAT3 pathway, including *ZNF431* [16], *IL-6R* [17], *IL6ST* [18], *ERBIN* [19], and *TGFBR* [20] gene defects, as well as CARD11 [21] deficiencies were categorized as HIES, expanding the genetic profile of HIES. In addition, the *DOCK8* deficiency [13], previously recognized as AR-HIES, is now considered as a combined immunodeficiency (CID) disease [15]. Patients with HIES exhibit eczema resembling atopic dermatitis features, but in more severe phenotype. Differentiation of HIES from severe atopic dermatitis (SAD) can facilitate early diagnosis and treatment.

#### 2.1.1. The Genetic and Clinical Spectrums of HIES

##### STAT3-HIES (OMIM147060)

AD-HIES (or STAT3-HIES), previously described as Job syndrome, was found to be caused by heterozygous mutations with DN effect in *STAT3* gene by Mingegishi et al. in 2007 [11]. STAT3 is a member of the STAT protein family and plays a key role in the signal transduction of many cytokines, such as interleukin (IL)-6, IL-10, IL-11 and IL-21 [22]. IL-6 is crucial for the differentiation of T-helper 17 (Th17) cells, which can contribute to the defense of extracellular bacterial and fungal pathogens by further secreting cytokines IL-17 and IL-22 [22]. STAT3 is also involved in B-cell differentiation and IgE production via IL-21 signaling [23,24]. IL-11 signaling, essential for the normal development of craniofacial bones and teeth, can restrict suture fusion and tooth number [25].

Patients with *STAT3* mutations can present with both immunological and non-immunological manifestations [11]. The clinical features of STAT3-HIES include early-onset eczema, recurrent skin infections and skin abscesses, recurrent pneumonia and resultant pneumatoceles, chronic mucocutaneous infection, along with skeletal, dental, and connective tissue abnormalities, such as characteristic facial features (a prominent forehead, deep-set eyes, broadened nasal bridge, and high-arched palate), retained primary teeth, bone fragility and scoliosis [11,26,27,28,29]. Early-onset (within the first week of life) papulopustular rash on the face and scalp is often the first present symptom and can develop to eczematous dermatitis within the first year. This chronic dermatitis is strongly associated with the colonization of *Staphylococcal aureus* [27,30]. Newborn rash, skin abscesses, chronic mucocutaneous candidiasis and pneumonia are highly specific of STAT3-HIES [27]. Arterial abnormalities predominantly with coronary artery ectasia or aneurysm [31], gastrointestinal manifestations like infection-related intestinal perforation [32], and malignancy hematopoietic in origin [26] have also been reported.

##### STAT3-Related Deficiencies

STAT3-related deficiencies share clinical phenotypes with STAT3-HIES due to the relevance of STAT3-dependent pathway. Here we classified ZNF431 deficiency (OMIM618282), IL-6R deficiency (OMIM618944), IL6ST deficiency (OMIM618523, 619752), TGFBR deficiency (OMIM609192, 610168), and ERBIN deficiency (OMIM606944) as STAT3-related deficiencies.

Homozygous loss-of-function (LOF) mutations of *ZNF341* gene in patients with HIES were first identified by Frey-Jakobs et al. in 2018 [33]. Zinc finger protein 431 (ZNF431) is a transcription factor that regulates the transcription of *STAT3* [16,33]. Patients with biallelic *ZNF431* mutations have low constitutive levels of *STAT3* mRNA and present with atopy, elevation of IgE, and susceptibility to *Candida* and *Staphylococcal aureus* infections, but they show mild skeletal or connective tissue abnormalities. Cutaneous manifestations involve dermatitis, pruritis, excoriated skin lesions, eczema, and skin infections particularly with *Staphylococcal aureus* [16]. Similar clinical features were also detected in patients with homozygous mutations in *IL-6 receptor* (*IL-6R*) gene by Spencer et al. in 2019 [17], manifesting as recurrent infections, abnormal acute-phase responses, elevated IgE, eczema, and eosinophilia. The binding of IL-6 and IL-6R further ligates glycoprotein 130 (GP130), leading to the phosphorylation of STAT3 and Janus kinase (JAK), as well as the nucleus transfer of STAT3 dimer [17].

The homozygous mutation of *IL6ST* was firstly identified in a patient as a novel immunodeficiency with phenotypic similarities to STAT3-HIES by Schwerd et al. in 2017 [18]. Then in 2020, DN *IL6ST* variants were described as another form of AD-HIES by Beziat et al. [34]. The patients have clinical features with greater similarity to that of STAT3-HIES, presenting with recurrent infections, bronchiectasis, eczema, high IgE, eosinophila, impaired acute-phase response, as well as skeletal and connective abnormalities (craniosynostosis and scoliosis) [34]. *IL6ST* encodes GP130, the co-receptor subunit for IL-6 family cytokines [18]. GP130 signaling is mediated via JAK/STAT3 pathway, accounting for the phenotypic overlap of DN *IL6ST* and *STAT3* mutations [18].

TGFBR deficiency, known as Loeys-Dietz syndrome, is caused by monoallelic mutations in *TGFBR1* and *TGFBR2* genes that encode for subunits of the transforming growth factor β (TGF-β) receptors. TGF-β signaling plays a crucial role in the immune system by regulating T-cell differentiation and regulatory T-cells (Tregs) function [35]. Patients with *TGFBR1* or *2* mutations showed defects of the SMAD2-dependent pathway and enhanced TGF-β signaling [36]. K. Felgentreff et al. reported in 2014 that patients with heterozygous mutations in *TGFBR1* and *TGFBR2* presented with immune dysregulation resembling STAT3-HIES, including highly elevated IgE, severe eczema, recurrent respiratory infections, skeletal and connective tissue abnormalities [20]. ERBIN deficiency caused by LOF mutations in *ERBB2-interacting protein* (*ERBB2IP*) was first described by J. Lyons et al. in 2017 [19]. It is an AD disorder that share similar clinical features with TGFBR deficiency. STAT3 can negatively regulate TGF-β signaling via the formation of a STAT3-ERBIN-SMAD2/3 complex. Therefore, the deficiency of ERBIN leads to the activation of TGF-β signaling [19]. These interactions may be the reason for the overlaps of their clinical manifestations.

##### DOCK8 Deficiency (OMIM243700)

Homozygosity or compound heterozygosity for deletions or mutations in *DOCK8* gene resulting in LOF protein were first identified by Zhang et al. in 2009 [14]. DOCK8 protein, a member of the DOCK180 superfamily of guanine nucleotide exchange factors, plays an important role in actin-cytoskeletal rearrangement, cellular migration and the formation of immune synapses [37]. Dendritic cells fail to migrate to local lymph nodes for T-cell priming in *Dock8* knockout murine model [38]. What is more, the DOCK8 deficiency leads to early death of T cells [39] and natural killer (NK) cells [40], which further prevents the generation of memory CD8+ T cells [41]. DOCK8 also regulates the activation of B cells and the generation of memory B cells, thus B cells from Dock8-deficient mice have difficulty in affinity maturation and antibody response [37]. Taken together DOCK8 deficiency have a broad effect on the immune system, characterized as a CID syndrome [14].

Apart from the classical manifestations of HIES like early-onset eczema, skin infections, pneumonia and elevated IgE, allergic manifestations including food allergies and asthma are more prevalent in DOCK8 deficiency than in STAT3 deficiency [42,43]. In addition, there is a susceptibility to cutaneous viral infections such as human papillomavirus (HPV), human simplex viruses (HSV), and molluscum contagiosum [43,44]. The poor control of virus infections further contributes to the higher risk of specific malignancy [44]. Progressive T-cell lymphoma and other cancers unrelated with viral infections have also been reported in DOCK8-deficienct patients [14]. Other reported manifestations include recurrent sinopulmonary infections, aortic and abdominal arterial vasculitis [45].

##### PGM3 Deficiency (OMIM172100)

PGM3 deficiency is caused by AR mutations of *PGM3* gene and described in affected patients with atopy, immunodeficiency, and neurological impairment by Zhang et al. and Stray-Pedersen et al. in 2014 [12,46]. PGM3 is a crucial enzyme in the synthesis of uridine diphosphate N-acetylglucosamine (UDP-GlcNAc), which is an important precursor for protein glycosylation. The pathogenesis of PGM3 deficiency resulting in HIES remains to be elucidated, but the inaccurate glycosylation of protein can lead to multisystem abnormalities, such as the impaired immune function and abnormal neuronal development [12,46]. Complete loss of Pgm3 causes embryonic lethality in mice [47], whereas *Pgm3* gene-trap mice can survive along with body weight loss, B-cell defects, and neurologic abnormalities [48].

PGM3 deficiency has variable clinical features ranging from the phenotype of STAT3 deficiency to severe combined immune deficiency (SCID) [46,49]. The patients present with atopic disease (eczema, allergy and asthma), recurrent skin and pulmonary infections, elevated IgE and eosinophilia, characteristic facial features (wide nostrils and prominent lips), skeletal abnormalities (scoliosis) [46,48]. Neonatal-onset SCID and neutropenia were reported in patients with deleterious *PGM3* mutations [46,50]. Notably, neurocognitive impairments occurred in early life can be detected in almost all patients, including development delay, low intelligence quotient, hypotonia, ataxia, dysarthria, sensorineural hearing loss, and myoclonus [12].

##### DN Mutations in CARD11 (OMIM 617638)

Caspase recruitment domain family member 11 (CARD11) is a scaffold protein and involved in lymphocyte receptor signaling [51]. Heterozygous mutations in *CARD11* with DN effect were first reported to cause CID and atopy features by Ma et al. in 2017, presenting with cutaneous viral infections, respiratory tract infections, early-onset moderate to severe atopic dermatitis, early-onset asthma and food allergy, elevated IgE and eosinophilia. Skeletal, dental and connective tissue abnormalities are absent [21,51].

#### 2.1.2. The Diagnosis of HIES

The clinical heterogeneity of HIES throws challenges for the differential diagnosis. The characteristic clinical manifestation and laboratory findings of each condition can help clinician to predict the underlying gene defect in HIES, and genetic analysis is essential for definitive diagnosis [28,52].

##### Clinical Manifestations

The atopic dermatitis-like skin lesions of HIES can be confused with the features of severe atopic dermatitis (SAD). Atopic dermatitis is prevalent with a fifth of affected population in developed countries [53]. The disease occurs during the first year of life (often after the 2 month of age) with a long clinical course of a relapsing-remitting nature [53]. Patients with SAD can also exhibit increased IgE and eosinophilia [54]. Cutaneous infections can be the secondary lesion of atopic dermatitis. The colonization of *Staphylococcal aureus* is the leading cause of skin and soft-tissue infections. Eczema herpeticum with HSV infection tends to occurs in SAD patients. Children are more susceptible to molluscum contagiosum virus infection [54].

There are some red flags for the hint of HIES, including early-onset eczematous skin lesion (prior to 2 months of life), recurrent viral, bacterial, or fungal infections, present of other affected family members, and other characteristic immunological manifestations [52,55]. Chronic mucocutaneous candidiasis (CMC) was significantly more prevalent in HIES compared to SAD, particularly in DOCK8-deficient patients [56]. STAT3-HIES is featured by skeletal and connective tissue abnormalities along with characteristic facial appearance [55]. Severe allergic symptoms, skin viral infections, and malignancies are more specific for DOCK8-deficient patients. They are more susceptible to common virus infections, including HSV and HPV. As for PGM3-deficient patients with variable manifestations, neurocognitive impairments can be peculiarly detected.

The National Institutes of Health (NIH) clinical scoring system [57] based on the presence or severity of 21 clinical and laboratory findings was established and developed specially for the identification of STAT3-HIES, suggesting with scores of at least 40 points. Woellner, C. et al. [10] proposed diagnostic guidelines for STAT3-deficient HIES in 2010, considering IgE > 1000 IU/mL, clinical features (recurrent pneumonia, newborn rash, pathologic bone fractures, characteristic face, and high palate), and lack of Th17 cells or a family history for definitive HIES, along with a DN heterozygous mutation in *STAT3* as the definitive diagnosis of STAT3-HIES.

##### Laboratory Findings and Genetic Analysis

Laboratory examinations including immunoglobulin levels, differential blood counts, as well as a lymphocyte subset analysis can be helpful during the process of diagnosis. Elevated serum IgE (often higher than 2000 IU/mL) and eosinophilia are the most typical laboratory findings [58]. For STAT3-HIES, immunoglobulin levels are usually normal, but a decreased percentage of Th17 cells (<0.3% of CD4+ T cells) in lymphocyte subset analysis can be a strong indication, especially for the differential diagnosis from atopic dermatitis [10,59]. In STAT3-associated disorders, decreased Th17 cell counts can also be detected. Lymphopenia, especially T-cell lymphopenia and less frequently B-cell lymphopenia, and low IgM serum levels are a diagnostic hallmark of DOCK8 deficiency [42]. Flow cytometry-based assays to detect the DOCK8 protein is a reliable method for its diagnosis [60]. PGM3 deficiency can present with lymphopenia and neutropenia. The T-cell lymphopenia affecting predominantly the CD4+ subset can result in reversed CD4:CD8 ratio [48].

Genetic analysis is necessary for the definitive diagnosis of HIES. The identification of the underlying gene mutations can be beneficial to disease managements and genetic counseling [58]. For those patients with suspected HIES, but lacking characteristic clinical and laboratory findings, genetic testing is essential to early diagnosis and appropriate treatment before the onset of complications.

#### 2.1.3. The Managements of HIES

Therapeutic options for HIES should be tailored to the type of mutation and the clinical manifestations of individuals. The prevention and treatment of infections of HIES patients is pivotal to the disease management [26,49]. Early diagnosis of HIES and timely implementation of antimicrobials enable vigilance of skin or pulmonary infections, further preventing the occurrence of complications, especially for STAT3-HIES patients who lack the classical signs of infection [8]. In addition, the management of atopic dermatitis can help to protect skin barriers and prevent viral or bacterial super-infection [61]. Emollients, topical steroids and topical tacrolimus can be applied, or oral steroids can be considered in severe cases.

Oral anti-staphylococcal antibiotics combined with conventional topical agents are recommended in STAT3-HIES patients because the recurrent skin infection of *Staphylococcal aureus* can exacerbate eczematous dermatitis. Antifungal therapy is also needed due to the susceptibility of CMC [8,26]. For the treatment of chronic lung diseases, antibiotic and antifungal, along with proper airway management are beneficial [52]. Immunoglobulin replacement can be strongly considered in patients with recurrent pulmonary infections. The employ of hematopoietic stem cell transplantation (HSCT) in STAT3 deficiency patients remains to be controversial [8,52].

The management of DOCK8 deficiency includes screening for and treatment of complications, immunoglobulin replacement, and definitive therapy with HSCT [42,62]. The potential infections should be evaluated and actively treated with prophylactic antimicrobials. Immunoglobulin prophylaxis is recommended based on impaired long-lived antibody responses in DOCK8-deficient patients [43]. HSCT has been the only curative treatment for DOCK8 deficiency, supported by multiple successful treatment cases [63,64]. HSCT is recommended at early stages of the disease, and the complications of infection or malignancy should be aggressively managed as well [8].

### 2.2. Wiskott-Aldrich Syndrome (WAS, OMIM301000)

#### 2.2.1. Inherited Pattern

WAS is a rare X-linked primary immunodeficiency disorder with an estimated incidence of 1:250,000 male birth [65]. *WAS*, as its pathogenic gene, was identified in 1994 by Derry et al. and located on the short arm of the X-chromosome (Xp11.23), encoding the Wiskott-Aldrich syndrome protein (WASp) [66]. In the Human Gene Mutation Database (HGMD) professional 2021.4, over 450 mutations have been detected, with missense mutations ranking as the first, followed by deletions mutations, splicing mutations, insertions, indels, and complex mutations [67].

#### 2.2.2. Molecular Mechanism

WASp is a member of the actin nucleation-promoting factors family with multiple functional domain, and predominantly expressed in hematopoietic cells [68]. WASp mainly remains in the resting state by autoinhibition in the cytoplasm. Upon activation, WASp can interact with Arp2/3 complex through its C-terminal VCA (verprolin homology domain, central hydrophobic region, acidic region) domain, thus mediating actin polymerization and cytoskeletal remodeling [68,69,70]. What is more, WASp also involves the transcription of *TBX21* gene, which encodes the key regulator of Th1 immune response [71].

Actin cytoskeleton is required for several cellular processes, including immunological synapse formation, cell motility and migration, autophagy and inflammasome regulation. Resultantly, loss of WASp activity leads to defect in hematopoietic and immune cell functions [68,72]. These deficiencies explain the abnormalities of both B- and T-cell functions, impaired monocyte chemotaxis, abnormal morphology of stimulated dendritic cells, as well as lymphopenia in patients of WAS [73]. The underlying mechanism of microthrombocytopenia is not fully understood. It is probably associated with the megakaryocyte dysfunction which leads to abnormal formed platelets [74]. Recent research proposed its correlation with friend leukemia integration 1 (FLI1), which can be induced by WASp depletion and play a crucial role in megakaryocyte differentiation [75].

#### 2.2.3. Clinical Manifestations

WAS has a large clinical spectrum characteristically associated with immunodeficiency, eczema, thrombocytopenia, a high incidence of autoimmunity and malignancies. The clinical presentation usually occurs after birth, presenting with bruises and spontaneous or prolonged bleeding due to low numbers of circulating platelets [70]. Eczema occurs in most affected patients during their first year of life. Bacterial infections (frequently *Streptococcus pneumoniae*, *Neisseria meningitidis*, and *Haemophilus influenzae*) manifesting as otitis media or pneumonia are common in WAS patients. They also have higher risks of viral infections and opportunistic infections [70,72,76]. Autoimmune manifestations including hemolytic anemia, vasculitis, IgA nephropathy, and inflammatory bowel disease have been reported [72,76]. Patients with WAS also have higher risks of malignancies with lymphoma and leukemia [72].

X-linked thrombocytopenia (XLT) is a relatively milder disorder caused by *WAS* gene mutations, with less problems in eczema and infections [69]. A 5-point severity score of WAS has been developed to differentiate XLT (score 0.5–3) from the classical WAS (score 3–5), on the basis of severity of thrombocytopenia, eczema, immunodeficiency and infections, and presence of autoimmunity or malignancy [69]. Genetic analysis and protein levels can help to differentiate XLT and WAS. According to the strong phenotype-genotype correlation, patients with mutations resulting in the absence or truncation of WASp are more likely to have the classical WAS phenotype, while patients with mutations that allowed the expression of normal-sized mutated WASp, often in reduced quantities, developed the XLT phenotype [73,77,78].

#### 2.2.4. The Diagnosis of WAS

The diagnosis of WAS should be considered in any boy with petechiae, easy bruising, spontaneous or prolonged bleeding, with or without eczema and/or recurrent infections, autoimmunity diseases, and/or malignancies [69]. The Laboratory examinations and genetic analysis can help to diagnose definitively.

Complete blood cell count and peripheral blood smear are necessary [76]. The reduced platelet count and platelet volume show favorable diagnostic value. However, the degree of thrombocytopenia is variable and sometimes lies in normal level, which can disturb the diagnosis [72]. Reduced platelet volume is pathognomonic for WAS/XLT, but mean platelet volume is difficult to measure in moderate to severe thrombocytopenia [76]. Peripheral blood smear demonstrating small platelets is more reliable in this condition [69].

Flow cytometry can investigate WASp expression in peripheral blood leukocytes, which predict the severity of clinical phenotype and can be a quick diagnostic indicator of WAS if the result shows a complete absence of WASp [79]. Genetic analysis is the gold standard for definitive diagnosis of WAS [69,76].

#### 2.2.5. The Managements of WAS

The management of WAS including supportive therapy and definitive therapy should be tailored to patients’ clinical manifestations and disease severity [69,72].

Once a child is diagnosed with WAS, definitive therapy should be considered, either HSCT or stem cell gene therapy (GT) [72]. Early definitive treatment is recommended for patients with absent WASp and *WAS* mutation consistent with classical WAS, regardless of their initial clinical presentation [72]. The HSCT for WAS patients has achieved excellent outcomes, with survival rates over 97% [80,81,82]. The post-transplant complications include graft-versus-host disease (GvHD), infections in the context of prior splenectomy, autoimmunity and infertility [72,80,81,82]. Gene therapy is a valuable therapeutic alternative of HSCT with lower risks of rejection reaction, GvHD, and infections [79]. Available data from recent GT clinical trials using lentiviral vectors supported the safety and efficacy of this therapeutic approach in the short- and medium-term, but information on the very long-term safety of GT (>10 years) is still limited [83,84,85].

Supportive therapy consists of the management of infection, thrombocytopenia, and autoimmune diseases before definitive treatment [72,76]. The employment of prophylactic antimicrobials and the strategies of anti-viral or anti-fungal medications should be decided based on the conditions of individuals [69]. It is recommended that all patients with classical WAS commence immunoglobulin replacement treatment after diagnosis [72]. Before definitive treatment, platelet transfusion is not supported in the absence of active bleeding due to the development of anti-platelet and anti-human leucocyte antigen antibodies after platelet transfusion [72]. The occurrence of autoimmunity can be managed with supportive care and immunosuppression [76]. For mild to moderate eczema, emollients, topical steroids and topical tacrolimus are recommended for the management. Oral steroids can be considered in severe cases [69,76].

### 2.3. Immune Dysregulation, Polyendocrinopathy, Enteropathy, X-Linked Syndrome (IPEX Syndrome, OMIM304790)

#### 2.3.1. Inherited Pattern

IPEX syndrome is a rare monogenic inborn error of immunity with the incidence below 1:1,000,000 [86]. It is a hemizygous disorder caused by the *forkhead box P3* (*FOXP3*) mutations firstly identified in 2001 and inherited predominantly in boys in an X-linked recessive manner. *FOXP3* gene is located in the centromeric region of chromosome (Xp11.23) and consists of 12 exons [86,87,88,89]. Over 90 mutations have been reported in HGMD, including missense/nonsense mutations, splicing mutations, small deletions and insertions [90].

#### 2.3.2. Molecular Mechanism

As a member of the forkhead/winged-helix family of transcriptional regulators, FOXP3 is encoded by *FOXP3* gene and acts as a DNA-binding protein during transcription that controls the development and function of Treg cells, thereby contributing to peripheral immune tolerance [91]. Patients with defect of FOXP3 show low-level Treg cells and elevated IgE levels, as well as higher rates of autoantibodies [89,92]. Impaired Treg cell function is associated with the development of autoimmunity, severe food allergy, and an unbalanced Th2 response [92,93]. The mice with mutations in *Foxp3* gene demonstrated similar phenotype with IPEX syndrome and multi-organ lymphocytic infiltration [94].

#### 2.3.3. Clinical Manifestations

The clinical features of IPEX syndrome are dominated by autoimmunity and characterized by a triad of enteropathy, polyendocrinopathy, and dermatitis [95]. Enteropathy associated with watery diarrhea often occurs within the first year of life, with the mean onset age of 7.8 months [96]. Insulin-dependent diabetes mellitus (IDDM) and thyroiditis are most commonly reported endocrinopathy and can be concurrent [89]. Other autoimmune disorders, including autoimmune hemolytic anemia, neutropenia, autoimmune gastrointestinal disorders and cutaneous disorders have also been reported [89,95]. Skin disorders are widespread, manifesting as eczema, exfoliative dermatitis, psoriasiform dermatitis, pemphigoid nodularis, cheilitis, onychodystrophy, or alopecia [88]. Life-threatening infections including sepsis, meningitis, peritonitis, and pneumonia occurred in over a quarter of IPEX syndrome patients, mostly infected by *Staphylococcus*, *Cytomegalovirus*, and *Candida* [88,89].

#### 2.3.4. The Diagnosis of IPEX

IPEX syndrome should be considered in male patients with enteropathy, dermatitis, autoimmunity, endocrinopathy. The combination of laboratory findings and genetic analysis are pivotal for diagnosis and differential diagnosis [86].

Elevated serum IgE level and eosinophilia are typical laboratory findings [88]. Flow cytometry can detect decreased Treg lymphocyte subset, while absolute lymphocyte counts and CD4+ and CD8+ lymphocyte subsets are usually normal [86]. A higher rate of autoantibodies mostly against enterocyte can be detected in patients with IPEX syndrome [89].

#### 2.3.5. The Managements of IPEX

The management of patients with IPEX syndrome can be divided into two strategies. The primary therapeutic approach is allogeneic HSCT. For those patients without HSCT, immunosuppressive therapy and supportive therapy are recommended [86,88].

HSCT is the only potentially curative therapy for IPEX syndrome [87]. Early HSCT before the development of organ damages seems to lead to better outcomes in patients. Two long-term follow-ups respectively reported a 10-year survival of 72.8% [88] and a 15-year survival of 73.2% [95] in patients who underwent HSCT. Complications of post-HSCT include acute or chronic GvHD, transplant-related toxicity, and multiple infections [95].

A broad spectrum of supportive therapies should be carried out at once, comprising the prevention and treatment of infections (e.g., prophylactic antibiotics), as well as the measurements for endocrine disorders (e.g., insulin and/or thyroid hormones) and autoimmune disorder (e.g., intravenous immunoglobulins) [87]. Immunosuppressive therapy, often a combination of systematic corticosteroids and immunosuppressive drugs including calcineurin inhibitors, rapamycin and some monoclonal antibodies, can lead to remission of autoimmunity and plays a crucial role for the management of initial acute manifestation. Nutritional support, either enteral or parenteral is necessary for patients receiving IS [95].

### 2.4. STAT5B Deficiency (OMIM618985, 245590)

#### 2.4.1. Inherited Pattern

STAT5B deficiency is considered as one of IPEX-like syndromes with similar phenotype [88,89]. The *signal transducer and activator of transcription 5B* (*STAT5B*) gene mutations have been described with both AD and AR inheritance by Kofoed et al. in 2003 [97] and Klammt et al. in 2018 [98], respectively. In HGMD, 19 mutations have been detected, including missense/nonsense mutations, deletions, insertions, and regulatory mutations [99].

#### 2.4.2. Molecular Mechanism

The protein STAT5B encoded by *STAT5B* is involved in homeostasis and maintenance of Treg cells [100,101], as well as terminal NK cell maturation and NK cell lytic synapse formation [102]. Therefore, STAT5B deficiency can impair T-cell homeostasis and natural NK-cell function [102]. What is more, JAK2-STAT5B pathway involves the signal transduction of circulating growth hormone (GH), which is important for normal human growth [103].

#### 2.4.3. Clinical Manifestations

The clinical presentation of STAT5B deficiency is featured by severe postnatal growth failure with growth hormone insensitivity (GHI) syndrome and insulin-like growth factor (IGF)-I deficiency [104]. Other manifestations include severe eczema, immune deficiency (e.g., recurrent infections), and autoimmune disease (e.g., juvenile idiopathic arthritis, autoimmune thyroiditis, idiopathic thrombocytic purpura), which are thought to be associated with Treg-cell dysfunction [100]. STAT5B deficiency with heterozygous mutation shows a milder clinical GHI without severe immune and pulmonary problems [98].

#### 2.4.4. The Diagnosis of STAT5B Deficiency

Patients should be suspected with STAT5B deficiency if they present with both growth disorders and immunological abnormalities.

Patients usually have normal levels of GH at baseline, and show elevated GH concentration after stimulation [100]. In contrast, the concentration of serum IGF-I remains to be low even upon administration of GH [100]. Elevated prolactin levels can be detected [104]. Immunological related examinations can show moderate lymphopenia with low numbers of NK and T cells, as well as elevated B cell populations and IgG levels [101].

#### 2.4.5. The Managements of STAT5B Deficiency

The managements of STAT5B deficiency have two sections according to its clinical presentations. One is therapies for postnatal growth failure, GH therapy is not ideal due to the GHI [97], while IGF-I therapy is presumed to be effective [100]. The management of immunological disorders is another crucial section, including treatments for infections and immunosuppressive therapies for autoimmune conditions.

### 2.5. The Atypical Complete Digeorge Syndrome

#### 2.5.1. Inherited Pattern

DiGeorge syndrome (DGS) is a congenital disorder mostly caused by a hemizygous deletion of chromosome 22q11.2, a frequent chromosomal 1.5–3 Mb deletion with an incidence of 1 in 4000–6000 livebirths [105,106]. Sulagna C, et al. proposed that the deletion was attributable to aberrant meiotic exchange event [107]. More than 35 genes are present within the deleted region, among which *TBXI* was identified to be a major genetic determinant [108].

#### 2.5.2. Molecular Mechanism

The mechanism of atypical complete DGS still remains unclear. It has been proposed that patients with atypical complete DGS have extrathymic differentiation and oligoclonal expansion of a small number of T cells [109,110]. Other hypothesis suggested that autoreactive T cells might result in impaired immune tolerance and defective immune regulation [111].

#### 2.5.3. Clinical Manifestations

DGS is a congenital malformation characterized by cardiac anomalies, hypoparathyroidism, and thymus hypoplasia or aplasia [112,113]. DGS can be divided into complete and incomplete DGS according to the phenotype of thymus abnormalities [114]. Patients with complete DGS are athymic and account less than 1% of all DGS patients [114]. The atypical complete DGS is a subset of complete DGS. The patients manifest with eczematous dermatitis, oligoclonal T-cell expansion, and lymphadenopathy [111,112,113,114,115]. The cutaneous manifestations are diverse, varying from diffuse erythroderma with scale formation to a greasy seborrheic dermatitis appearance [115].

#### 2.5.4. The Diagnosis of Atypical Complete DGS

Cutaneous manifestations, lymphadenopathy, along with laboratory findings of an oligoclonal T-cell population in the peripheral blood can help for early diagnosis [115]. Unique histopathological findings contribute to differential diagnosis, with characteristics of satellite cell necrosis and dyskeratosis, as well as brisk peri- and intra-eccrine inflammation [115]. Analysis of the immunophenotype and T cell receptor Vβ repertoire of T lymphocytes help to determine whether the patient is athymic [116,117], further discriminate atypical complete DGS from other conditions such as partial DGS or Omenn syndrome.

#### 2.5.5. The Managements of Atypical Complete DGS

Immunosuppressive therapy is required for atypical complete DGS [110,111]. Thymic transplantation can correct severe immunodeficiency. M. Louise, et al. reported the 1-year survival rate of 73% in 50 infants with complete DGS after transplantation of thymus tissue. Most deaths occurred prior to development of T cells due to severe infection [118]. They also reported the efficiency of thymus transplantation with immunosuppression in six infant patients. Among them, four patients with atypical complete DGS received immune reconstitution as polyclonal T-cell populations developed in 1 year after transplantation [119]. The reported adverse events after transplantation include hashimoto thyroiditis, autoimmune hepatitis, cytopenias, enteritis/colitis, and autoimmune hepatitis [119,120].

### 2.6. Omennsyndrome. (OS, OMIM603554)

#### 2.6.1. Inherited Pattern

OS is in a genetically heterogeneous condition with a frequency of 1:5,000,000 [121]. It was first confirmed to be related with LOF mutations in *RAG1* and *RAG2* genes [122,123]. Up to date, *DCLRE1C* [124], *RMRP* [125], *IL7RA* [126] and *IL2RG* genes are linked to OS.

#### 2.6.2. Molecular Mechanism

All pathogenic genes associated with OS impede the formation and maturation of immunology system. Take RAGs as an example. A process termed V(D)J site-specific recombination takes responsibility of diversity of the immune repertoire which is generated by somatic assembly of the antigen receptor variable gene segments. The specific binding is essential to this conserved recombination. RAG1/RAG2 dysfunction impedes the initiation of V(D)J recombination and leads to complete absence of mature B and T cells [127,128].

#### 2.6.3. Clinical Manifestations

OS is in SCID condition characterized by neonatal diffuse erythematous/eczematous rash, exfoliative dermatitis with dry and flaky skin, hypotrichosis, lymphadenopathy, hepatosplenomegaly, repeated infections, eosinophilia and elevated IgE [129,130]. Patients would frequently present with chronic diarrhea and pneumonitis in the first year of life and then failure to thrive [131,132]. Without proper treatment, OS shows a high mortality.

#### 2.6.4. The Diagnosis of OS

Cutaneous biopsy shows that early skin biopsy is beneficial to diagnosing OS. Key histopathological pathological features include epidermal hyperplasia, necrotic keratinocytes, acanthosis and parakeratosis and dermal lymphocytic infiltrate (CD3+) [133]. Abnormal expansion of T-cell clones in peripheral blood and tissues, along with a low number of CD19+ B cells are also the characteristic markers. Genetic analysis gains the concise diagnosis of OS.

#### 2.6.5. The Managements of OS

Outcomes of OS are greatly improved by early diagnosis and treatment. As the first-line therapy, HSCT shows fine improvement in survival rates [134]. Additionally, immunosuppressive drugs including prednisone and cyclosporin A are introduced to treat OS patients [131].

### 2.7. X-Linked Agammaglobulinemia (XLA, OMIM300755)

#### 2.7.1. Inherited Pattern

XLA is an immunodeficiency characterized by maturational arrest of B cells development with the incidence of 1:100,000–200,000. XLA, the most common genetic agammaglobulinemia, comprises 85% of subjects of agammaglobulinemia [135]. *Bruton tyrosine kinase* (*BTK*), the affected gene in XLA, was firstly discovered in 1993 by S Tsukada [136] and D Vetrie [137]. It is reported that missense mutations accounting for the most common mutation type in *BTK*, followed with insertion or deletions, nonsense mutations, splice site mutations, and large deletions [138,139]. Agammaglobulinemia with other inherited pattern were also concluded: AR agammaglobulinemia was associated with mutations in several other genes such as *IGHM*, *IGLL1*, *CD79A*, *CD79B*, *BLNK*, *PIK3R1*, and *TCF3* genes; AD-related agammaglobulinemia was also found with *LRRC8A* and *TCF3* gene mutations [135].

#### 2.7.2. Molecular Mechanism

BTK, expressed mainly in hematopoietic cells, is a non-receptor kinase that plays a crucial role in both B cell development and function of mature B cells [140]. Through the interaction of its pleckstrin homology domain with phosphatidylinositol-3,4,5-triphosphate (PIP3), BTK is transiently recruited to the membrane. After phosphorylated by SYK or SRC family at Y551 site, BTK autophosphorylates at Y223, which stabilizes the active conformation and fully activates BTK kinase activity. BTK is essential to B cell receptor signaling, B cell development in bone marrow and B cell malignancies. Alterations in the BTK can lead to primary B cell immunodeficiency and humoral immunodeficiency with decreased or diminished immunoglobulin production [141].

#### 2.7.3. Clinical Manifestations

Due to a severe block of B cell development in the bone marrow, decreased B cells in the circulation and almost completely absent antibodies in the serum are frequently discovered in XLA patients. Resultantly, recurrent infections including respiratory infections, otitis, sinusitis, skin infections, cervical lymphadenitis, epididymitis, osteomyelitis, urinary tract infection, gastroenteritis and chronic diarrhea, invasive infections (sepsis, meningitis, encephalitis), arthritis and hepatitis are described [139].

Cutaneous manifestations in XLA mainly present with severe skin infections like chronic skin ulcers, refractory cellulitis, skin abscesses, skin edema and discoloration and folliculitis [142,143]. Wheal and erythema, typical atopic dermatitis, and skin cancer were also reported in XLA patients [144,145,146].

#### 2.7.4. The Diagnosis of XLA

A male with paucity of lymphoid tissue and clinical history such as recurrent infections should be noted. Laboratory findings include: (1) marked reduction of serum immunoglobulins including IgG, IgM and IgA; (2) markedly reduced numbers of CD19-/CD20-expresssing B lymphocytes in the peripheral circulation; (3) low antibody titers to vaccine antigens; (4) severe neutropenia. However, assessment of BTK and family history of XLA are mandatory for the diagnosis of XLA [147,148].

#### 2.7.5. The Managements of XLA

XLA patients should be treated with replacement immunoglobulin and prophylactic antibiotics to prevent infections. Intravenous immunoglobulin (IVIG) at intervals of 2–4 weeks or subcutaneous immunoglobulin (SCIG) at intervals of 1–14 days should be used and adjusted by IgG level associated with clinical course. It is reported that increased IgG level is beneficial to decreased pneumonia incidence in patients with primary immunodeficiency [149,150].

## 3. Inherited Metabolic Diseases

### 3.1. Acrodermatitis Enteropathy (AE, OMIM201100)

#### 3.1.1. Inherited Pattern

Being a rare AR disorder, AE was first described by Brandt et al. in 1936 [151] and confirmed to be related with LOF mutations in *SLC39A4* gene, which codes the zinc (Zn) transporter ZIP4 [152,153]. In the HGMD, a total of 56 mutations have been described including: missense/nonsense, and splicing site mutations; small insertions/deletions; and few gross rearrangements [154]. Inherited AE occurs worldwide with an estimated incidence of 1 per 500,000 children and has no apparent preference for race or sex [155].

#### 3.1.2. Molecular Mechanism

Zn is stably maintained in the weight of 2–3 g in a human body. In all organs, skin is the third most Zn-abundant tissue while skeletal muscle, bones, liver and skin contains 60%, 30%, 5% and 5% respectively [156]. Flawed function of ZIP4 leads to disability of Zn absorption within the gastrointestinal tract and causes Zn deficiency. Compromised immunity is one of the first signs of zinc deficiency and the role of zinc in the immune system is known for a firm molecular basis [157].The skin inflammation pattern of AE is essentially similar to contact dermatitis induced by multiple irritants in dietary zinc-deficient mice [158]. As the particular locations which are primarily exposed to external irritants, perioral, acral and anogenital areas seem to reasonably present typical lesions [158].

#### 3.1.3. Clinical Manifestation

AE patients often suffer from acral and perioral dermatitis, alopecia, and diarrhea [159,160,161,162]. For the reason that breast milk usually contains adequate Zn with a high concentration (>3 mg/L) but progressively declines to <1 mg/L by 6 months [163], typical clinical manifestations frequently present at the time of weaning from breast or formula feeding. However, the complete symptoms are seen only in one-third of patients [164].

Cutaneous manifestations of AE consist of sharply demarcated eczematous or psoriasiform plaques, which symmetrically start with perioral and retro-auricular sites and spread to extremities. Vescicolo-bullous, pustular, and erosive lesions may also develop in the sites of mouth, eyes, noses, the scalp and perineum. Nail, oral and ocular disorders may occur [165]. Additional features could be loss of appetite, irritability and apathy; development retardation, testicular atrophy; neuropsychiatric features, hyposmia and hypogeusia [166]. Without proper management, extensive erosions associated with a predisposition for fungal (especially *Candida albicans*) or bacterial (e.g., *Staphylococcus aureus*, *Pseudomonas aeruginosa* [164]) colonization may follow.

#### 3.1.4. The Diagnosis of AE

Skin histological findings of AE are nonspecific [167,168,169]. The most common findings are alternating orthokeratosis and parakeratosis. Decreased stratum granulosum, acanthosis and focal acantholysis could also be seen. Dilated capillaries and sparse lymphocytic infiltration in the papillary dermis are the subsequent findings. Secondary skin lesions may present the characteristic of the bullae, while chronic lesions sometimes show a psoriasiform pattern [164,169,170].

Fasting Zn levels of less than 70 μg/dL (10.71 μmol/L) or post-meal levels of less than 65 μg/dL (9.95 μmol/L) are indicative for Zn deficiency. However, low Zn levels in plasma or serum, which only accounts for 0.1% in the whole body, do not directly indicate Zn deficiency [167], which indicates the importance of *SLC39A4* gene analysis. Additionally, Zn-dependent enzymes, such as alkaline phosphatase, can be measured as a hallmark [171].

#### 3.1.5. The Managements of AE

Oral administration of zinc sulfate is the base treatment for AE. Initial Zn dose of 5–10 mg/kg/d and maintenance doses of 1–2 mg/kg/d are recommended [170]. Clinical improvement can be observed after just a few days, whereas hereditary AE requires life-long replacement therapy [172]. However, the interruption of treatment could inevitably lead to relapses and the cutaneous manifestations are the first to recur [165].

### 3.2. Multiple Carboxylase Deficiency

Biotin, an water-soluble vitamin, is necessary for the activation of carboxylases and crucial in glucose, amino acid and fatty acid metabolism [173]. Two metabolic syndromes with decreased biotin, caused by disorders of holocarboxylase or biotinidase synthetase, are referred to multiple carboxylase deficiency [174]. Age of onset is used to differentiate between holocarboxylase synthetase (HLCS) deficiency and biotinidase (BT) deficiency. HLCS deficiency frequently occurs hours to weeks of birth while biotinidase deficiency generally presents after 3 months [175]. however, there are exceptions for both disorders.

#### 3.2.1. Holocarboxylase Synthetase Deficiency (HLCSD, OMIM253270)

##### Inherited Pattern

HLCSD, which typically presents at birth or affects children below the age of 2 months, is a rare AR inborn error of the biotin metabolism. The estimated incidence of the disease is less than 1 in 200,000 live births. Y Suzuki, et al. cloned the human *HLCS* cDNA and mapped *HLCS* gene to chromosome 21q22.1 in 1994 [176]. In the HGMD, a total of 64 mutations have been described including: missense/nonsense, and splicing site mutations; small insertions/deletions; and few gross deletions and complex rearrangements [177]. And there is a relationship between clinical biotin responsiveness and the residual activity of HLCS [178].

##### Molecular Mechanism

Since the early part of the last century, biotin has been recognized as an essential nutrient. Biotin requirement is fulfilled in through diet, endogenous reutilization of biotin and capture of biotin generated in the intestinal flora [179]. However, biotin deficiency is associated with protein malnutrition [180], and marginal biotin deficiency in pregnant women may be teratogenic [181].

Free-form biotin can directly enter the biotin pool and is used to convert four carboxylases from the inactive to the active form. HLCS takes the responsibility for covalently linking biotin to four carboxylases: propionyl-CoA carboxylase (PCC), 3-methylcrotonyl-CoA carboxylase (MCC), pyruvate carboxylase (PC) and acetyl-CoA carboxylase (ACC) [182,183]. Failure to approach biotin causes reduced activity of these carboxylases and leads to multiple carboxylase deficiency. *Hlcs*-knockout mice is embryonically lethal in both early stages and midway through pregnancy due to a depletion of biotinylated carboxylases [184].

##### Clinical Manifestations

The typical symptoms of HLCSD manifest as metabolic acidosis (emesis, hypotonia, lethargy, seizures, hyperammonemia, tachypnea and coma). Cutaneous lesions present with localized erythematous dermatitis [185], psoriasis-like dermatitis [186], periorificial and intertriginous dermatitis [187], keratoconjunctivitis and nonscarring diffuse alopecia with loss of hair luster. The erythema is well defined around the eyes, nose, mouth and on the perineum. Other symptoms such as feeding problems, a variety of central nervous system abnormalities and developmental delay could also be seen in HLCSD.

##### The Diagnosis of HLCSD

Cutaneous histopathology shows superficial perivascular lymphocytic infiltration with regular acanthosis, hyperkeratosis and parakeratosis and hypogranulosis [187], which is nonspecific.

HLCSD diagnosis is suggested by blood enzymatic determination, urine organic acids and verified by *HLCS* gene variants [188]. Tandem mass spectroscopy analysis of analytes such as amino acids, acylcarnitines and multiplex enzyme analysis on dried blood spots are the mainstay of testing for HLCSD. The detection of elevated hydroxypentanoylcarnitine (C5-OH) could be screened for HLCSD. Additionally, urine organic acid profile may demonstrate elevated lactic, 3-OH isovaleric, 3-OH propionic, methylcitric, and tiglylglycine consistent with LOF biotin-attached carboxylases [183]. However, biotinidase and zinc levels are normal.

##### The Managements of HLCSD

Without early diagnosis and proper treatment, HLCSD is associated with high morbidity and mortality [189]. However, prompt biotin supplement is highly associated with regression of the disease and the fine clinical outcomes, especially before antennal stage [190]. Oral biotin is the effective treatment and the doses ranging from 3 to 200 mg/day [191,192]. The metabolic disorders could be corrected within 2 days to 2 weeks and continued oral biotin therapy is essential to the improvement of the prognosis [183].

#### 3.2.2. Biotinidase Deficiency (BTD, OMIM253260)

##### Inherited Pattern

Mutations in the *BTD* gene produce the AR disorder, BTD, known as the late-onset MCD whose incidence is 1 in 60,000 births [193,194]. 284 variants of *BTD* that alter BT activity have been reported so far. All types of variants have been observed: missense/nonsense, splicing, regulatory, small insertion/deletion/indel, gross deletion and complex rearrangement [195]. Six most severe and common pathogenic allelic variants of *BTD* are founded: c.98_104delinsTCC, c.511G > A and c.1330G > C, c.1612C > T, c.1368A > C, c.1330G > C [196].

##### Molecular Mechanism

BT is the enzyme that recycles the biotin [197]. Dietary protein-linked and carboxylase-bounded biotin must be degraded to release biocytin. Small biotinyl-peptides which could be further cleaved by BT, release the free-form biotin to the pool [198]. Patients with inherited BTD are unable to utilize the biotin in food or recycle the biotin in carboxylase turnover.

##### Clinical Manifestations

The clinical presentation of BTD includes hypotonia, seizures, organic aciduria, mild hyperammonemia, feeding problems, developmental delay and breathing problems such as hyperventilation, laryngeal stridor, and apnea [199]. Cutaneous manifestation exhibits the following symptoms: eczematous skin rash and palmoplantar keratosis, diffuse alopecia or fragile hair [200], and recurrent viral or fungal infections. Seborrheic dermatitis-like eruptions and scaly erythematous plaques over the flexors and perioral areas can be detected [201,202]. The secondary lesions such as crusting, lichenification, and open lesions can be seen [203]. Some patients develop neurological disorders, involving ataxia, mental retardation, hearing loss, optic atrophy, myelopathy, and Leigh syndrome [204]. Symptoms of untreated BTD frequently appear between the ages of 1 week and 10 years, with a mean age of 3.5 months [205].

##### The Diagnosis of BTD

Quantitative determination of BT enzyme activity in serum/plasma with *BTD* genetic analysis is the golden standard to diagnose the BTD. Other biochemical abnormalities such as metabolic ketolactic acidosis, hyperammonemia, organic aciduria and elevated carnitine (in plasma and/or urinary) are also useful to identifying BTD [199]. Individuals with severe BTD have lower than 10% mean normal serum enzyme activity, while partial BTD patients have 10–30% of mean normal serum BT activity [206]. However, the dermatopathology shows no clinical specificity.

##### The Managements of BTD

Excluding the neurological abnormality, all clinical and biochemical manifestations of BTD can be alleviated or reversed with biotin (5–20 mg/day) [197]. As the child grows, the dosage of biotin decreases. The index of urine organic acid is used to estimate whether the biotin is enough [207].

### 3.3. Prolidase Deficiency (PD, OMIM170100)

#### 3.3.1. Inherited Pattern

PD is a rare AR multisystem disorder associated with *PEPD* mutations, which is verified by A Tanoue in 1990 [208]. The estimated occurrence of PD is 1 case in 2,000,000 births [209]. More than 90 cases are reported with 42 *PEPD* variants [210].

#### 3.3.2. Molecular Mechanism

Prolidase, a cytosolic manganese-dependent peptidase, is the only enzyme which hydrolyzes the tertiary amide bond involved in imidodipeptides. So prolidase performs crucially in protein synthesis and degradation, especially of proteins rich in iminopeptides such as fibrillar collagens [211,212].

#### 3.3.3. Clinical manifestations

Clinical symptoms of PD patients show a wide range from mild to severe. Cutaneous lesions such as refractory ulcerations, telangiectasis, impetigo-like eruptions, necrotic papules, premature graying of the hair, photosensitivity, erythematous maculopapular rash, and hypertrichosis can be observed in PD [213]. Anaphylaxis to multiple food allergens, allergic rhinitis, and asthma, shows severe atopy [214]. Other symptoms include bone disorders, mental retardation, respiratory infections and facial dysmorphisms, autoimmunity, and splenomegaly [215].

#### 3.3.4. The Diagnosis of PD

For diagnosing PD, methods are described as followed: (1) based on the determination of enzyme activity in leukocytes, erythrocytes, and skin fibroblast culture; (2) elevated levels of imidodipeptids in uria; (3) *PEPD* variants with classical clinical symptoms [216]. Skin biopsy found nonspecific changes including slight hyperkeratosis, edema, and parakeratosis with neutrophil leukocyte and lymphocyte infiltration [213].

#### 3.3.5. The Management of PD

The treatment of PD is symptomatic and has no curative regimen. Topical treatments including proline, GH, and tacrolimus, are used to replace prolidase or stop ulcerative progression [217,218,219]. Usage of systemic immunosuppressive medications or packed red blood cells transfusions have been also reported [220,221].

## 4. Rare Syndromes

### 4.1. Severe Dermatitis Multiple Allergies and Metabolic Wasting (SAM) Syndrome (SAM Syndrome, OMIM615508)

#### 4.1.1. Inherited Pattern

SAM syndrome is a rare genodermatitis caused by recessive homozygous or compound heterozygous LOF mutations in *desmoglein 1* (*DSG1*) gene, or dominant heterozygous mutations in *desmoplakin* (*DSP*) gene [222,223]. Since Samuelov L et al. first found the *DSG1*-related SAM syndrome [222], a total of 16 individuals were reported.

#### 4.1.2. Molecular Mechanism

Desmoplakin and desmogleins form the integral part of desmosomes, which serves as an anchor for adjacent epithelial cells to link to one another. Proteins in desmosomes play a role in cell signaling and skin barrier function [224].

#### 4.1.3. Clinical Manifestations

SAM syndrome presents with a broad spectrum of skin phenotypes and multi-system manifestations. Cutaneous symptoms include erythroderma, palmoplantar keratoderma, and ichthyosis. Besides, pruritus, hypotrichosis, woolly hair, and nail abnormalities are common in SAM syndrome. Other disorders such as food sensitization, dental abnormalities, developmental delay, recurrent infections, elevated IgE, eosinophilia, ophthalmic abnormalities cardiac abnormalities, gastrointestinal problems, brain abnormalities, can also be observed in SAM syndrome [223,225,226,227,228].

#### 4.1.4. The Diagnosis of SAM Syndrome

Skin histopathology shows psoriasiform hyperplasia and hyperkeratosis, which presents no specificity. Elevated eosinophils, reduced 25-hydroxy-vitamin D and elevated IgE could be detected in SAM syndrome while all of these could not be used as the diagnosis standards. Genetic analysis with the typical clinical manifestation is the authoritative diagnostic criteria for SAM syndrome [228].

#### 4.1.5. The Managements of SAM Syndrome

No proper treatment is recommended to the SAM syndrome. However, several groups made novel trials: *DSP*-related SAM syndrome performed a favorable response to ustekinumab [229]; oral acitretin and topical pimecrolimus released cutaneous eruption and oral gabapentin relieved pruritus [230]; successful treatment with secukinumab in SAM syndrome and SAM-like syndrome were also reported [231].

### 4.2. Netherton Syndrome (NS, OMIM256500)

#### 4.2.1. Inherited Pattern

NS is a rare, multisystemic, AR genodermatosis with an incidence of one patient in 200,000 newborns, which is also thought to be the cause of up to 18% of congenital erythrodermas. It was first depicted by Comel in 1949 [232] and Netherton in 1958 [233]. So far, a total of 108 variants are found, which involve all types of mutations [234]. In addition, nine lethal variants are depicted: c.153delT, c.238insG, c.375_376delAT, c.C649T, c.997C > T, c.1111C > T, c1431–12G > A, c.715insT, and 375delAT [235].

#### 4.2.2. Molecular Mechanism

*SPINK5* (*serine protease inhibitor of Kazal type 5*), localized on chromosome 5q32, encodes LEKTI (lympho-epithelial Kazal type related inhibitor), a multidomain serine protease inhibitor expressed in the thymus and epithelia [236]. Deficiency in LEKTI causes the increased activity of the epidermal serine proteases kallikrein 5 (KLK5), which degrades DSG1, leading to epidermal desquamation and altered skin barrier function [237]. Hyperactivity of KLK5 could increase the expression of thymic stromal lymphopoietin (TSLP), which elevates levels of IgE [238].

#### 4.2.3. Clinical Manifestation

The clinical manifestation of NS is characterized by a triad of trichorrhexis invaginata, icthyosis linearis circumflexa (ILC), and an atopic diathesis. ILC performs as pruritic erythematous and serpiginous plaques with double-edged desquamation showing polycyclic and serpiginous borders. The recalcitrant intensely pruritic dermatosis frequently comes with hair abnormalities, such as bamboo hairs (trichorrhexis invaginata), golf tee, and matchstick hairs. Atopic dermatitis, eczema-like eruptions, pruritus, asthma, allergic rhinitis, angioedema, elevated IgE, and/or hypereosinophilia and sensitivity to multiple allergens are included in the atopic diathesis [239]. Other symptoms present as a mental deficiency, neurologic disorders, development delay, recurrent infections, aminoaciduria, and hypergammaglobulinemia [240,241].

#### 4.2.4. The Diagnosis of NS

Skin biopsy usually exhibits hyperkeratosis, acanthosis, focal hypergranulosis, and superficial perivascular lymphocytic infiltration, which is apparently unspecific [242]. Hair shaft abnormality could be uneasy to find because only 20–50% of hairs are affected. Laboratory findings are significant in increased peripheral eosinophilia and elevated IgE levels [240]. Finding gene mutations in *SPINK5* is essential to diagnosing NS.

#### 4.2.5. The Managements of NS

NS could be confused with atopic dermatitis but shows an unsatisfactory response to topical corticosteroid treatment. However, topical tacrolimus and pimecrolimus or IVIG show good effects in NS treatment [241,243]. UVB treatment could also lead to positive improvement of lesions [244]. Oral retinoids are also a nice try to treat NS patients [245].

### 4.3. Peeling Skin Syndrome Type B (PSS-B, OMIM 270300)

#### 4.3.1. Inherited Pattern

Generalized PSS (peeling skin syndrome) has been subclassified into a noninflammatory type (peeling skin syndrome type A), and an inflammatory type (PSS-B) [246]. PSS-B, a rare AR genodermatosis, results from LOF mutations in the *CDSN* gene, which was first found in 2010 by Vinzenz Oji et al. [247]. So far, a total of 18 individuals with 16 variants are reported, including missense/nonsense, splicing, small deletions or insertions, and gross deletions [248].

#### 4.3.2. Molecular Mechanism

Corneodesmosin (CDSN), an extracellular component of corneodesmosomes, locates in the corneodesmosomal core and is covalently linked to the cornified envelope of corneocytes. CDSN is highly expressed in hair follicles and cornified epithelia, and plays an important role in maintaining desmosome integrity, the proper development and function of the skin barrier, and normal hair follicle formation [249,250]. Cdsn deficiency mice showed stratum corneum detachment resulting from abnormal desmosome formation [251].

#### 4.3.3. Clinical Manifestations

Cutaneous symptoms of PSS-B present with generalized, pruritic scales and ichthyosiform erythroderma which begin at birth and evolve to mild erythematous areas worsening in summer, with recurrent skin infections and vesiculations. Along with hypotrichosis, hairs of PSS-B patients can be easily plucked. Nail abnormalities include onychodystrophy and white nail changes [247,252,253,254,255,256,257]. Other symptoms including food allergies, repeated episodes of angioedema, urticaria and/or asthma, feeding difficulty, failure to thrive, hypereosinophilia and hypoproteinemia, and microadenoma of the pituitary gland, nephrocalcinosis with hypercalciuria, dysphonia, and intellectual disability can be observed in PSS-B [258].

#### 4.3.4. The Diagnosis of PSS-B

Histopathology reveals the extensive subcorneal cleavage in the epidermis with irregular acanthosis [247]. Elevated IgE and *CDSN* gene analysis are important clues to diagnosing PSS-B [259].

#### 4.3.5. The Managements of PSS-B

Ustekinumab [258] and low-potency topical corticosteroid ointment, oral retinoids fail to alleviate the skin lesions, while topical 0. 005% calcipotriol ointment could reduce skin peeling and erythema [260]. PSS can usually be well managed with topical treatments and hygienic measures alone, and it seems to improve with age [259].

## 5. Conclusions

**Figure 1:**
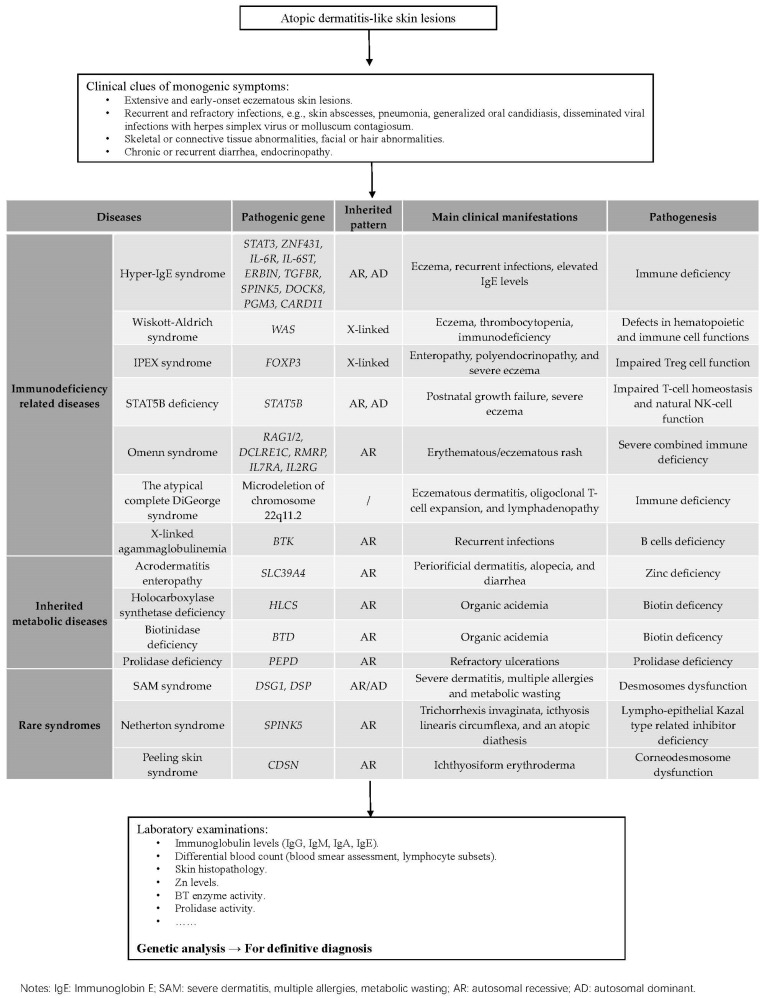
How to distinguish diseases performed with atopic dermatitis-like lesions.

A topic dermatitis-like lesions can frequently present in patients with genetic diseases. In this review, three main categories including immunological disorders, metabolic diseases, and rare syndromes are discussed. We have presented the diagnostic clues that simplify the differential diagnosis, especially for immunological disorders, shown in Figure 1 and Table 1.

The clinical indications of an underlying PIDD conclude severe and/or early-onset eczema, features of immunodeficiency. Notably, the phenotypes of PIDDs and atopic dermatitis partially overlap, presenting with increased serum IgE levels, eosinophilia, and eczema lesions, which indicates the shared immune pathways. Indeed, atopic dermatitis is an inflammatory skin disease caused by multiple factors, among which dysregulated type 2 immunity driven by Th2 cells plays a crucial role. Here, HIES including STAT3 deficiency and STAT3-related deficiency is also associated with activated Th2 responses and decreased Th17 generations; CARD11 deficiency, IPEX syndrome, STAT5B deficiency, and OS affect Treg diversity or function that regulates Th2 immunity. WAS and DOCK8 deficiency are associated with impaired actin assembly and immunological synapse formation, and XLA shows B cells deficiency. These discoveries provide additional insights into the pathogenesis of atopic disorders. For the diagnosis of PIDDs with atopic dermatitis-like lesions, laboratory tests that consist of blood counts, lymphocyte subsets count and serum immunoglobulin levels are recommended for suspected patients. Low lymphocyte counts can be indicative of OS, XLA, or DOCK8 deficiency, and WAS is supported if showing low mean platelet volume. Flow cytometric analysis is a valuable tool especially for identifying STAT3-HIES that low Th17 cell counts can point toward STAT3 related disorders. However, assessment of genes and family history of affected subjects are mandatory for differentiation.

As for metabolic genodermatitis, indicators in metabolism show great importance: Zn deficiency is associated with the phenotype of perioral and perianal dermatitis, diarrhea highly suggests AE resulting from *SLC39A4* mutations; organic acidemia reminds us to pay attention to MCD related to *BT* and *HLCS* variants; reduced prolidase enzyme activity and elevated levels of imidodipeptids hint us with *PEPD*-associated PD.

Apart from all the immunologic and metabolic diseases, rare syndromes should always be taken into consideration in diagnosing eczema-like dermatitis. However, it is so hard to distinguish rare syndromes due to their complex clinical manifestations. Genetic analysis provides us with clear diagnosis clues: SAM syndromes are linked with *DSG1* and *DSP* mutations; NS is due to LOF mutations in *the SPINK5* gene; *CDSN* variants are always detected in PSS-B patients.

The recognition of these pathogeneses shed new light on clinical and mechanistic associations among allergy, metabolism, and desquamation. Comprehensive knowledge of the regulation of immunological, metabolic, and keratinization functions and the potential therapeutic agents are depicted. Emphatically, genetic analysis is still crucial and essential for definitive diagnosis. Early diagnosis allows for prompt and specific treatments prior to the onset of complications. Aside from the direct influence on the affected subject, research on the atopic dermatitis-like syndrome not only broadens our understanding of human biology but also promotes development in the management of common conditions.

## Figures and Tables

**Table 1:** The genetic and clinical spectrums of hyper IgE syndromes.

Disease	Pathogenic Gene	Inherited Pattern	Immunological Features		Clinical Manifestations	
			Lymphocyte Subsets	Immunoglobulin Levels	Immunological Manifestations	Non-Immunological Manifestations
STAT3-HIES (STAT3 deficiency)	*STAT3*	AD LOF(DN)	T lymphopenia, decreased Th17 cells, reduced memory B cells	Highly elevated IgE	Early-onset eczema, bacterial skin and respiratory infections, abscesses, mucocutaneous candidiasis, pneumonias leading to pneumatoceles	Distinctive facial features, skeletal and connective abnormalities (retained primary teeth, bone fragility, hyper-extensive joints, and scoliosis), Arterial abnormalities
ZNF431 deficiency	*ZNF431*	AR	Decreased Th17 cells and NK cells, reduced memory B cells	Elevated IgE and IgG	Phenocopy of STAT3-HIES	Mild facial features, mild skeletal and connective abnormalities (hyper-extensive joints, scoliosis)
IL-6R deficiency	*IL-6R*	AR	Low switched memory B cells	Highly elevated IgE, normal/low IgM, G, A	Severe eczema, skin abscesses, pneumonia	Mild skeletal and connective abnormalities (scoliosis)
IL6ST deficiency	*IL-6ST*	AR, AD LOF(DN)	Decreased Th17 cells, reduced memory B cells	Elevated IgE	Phenocopy of STAT3-HIES	Facial features, skeletal abnormalities (craniosynostosis and scoliosis)
ERBIN deficiency	*ERBB2IP*	AD	Increased circulating Treg	Elevated IgE	Eczema, recurrent respiratory infections	Skeletal and connective tissue abnormalities like STAT3-HIES
Loeys-Dietz syndrome (TGFBR deficiency)	*TGFBR1,* *TGFBR2*	AD	Normal	Elevated IgE	Eczema, recurrent respiratory infections, Allergy	Aortic aneurysms, skeletal and connective tissue abnormalities like STAT3-HIES
DOCK8 deficiency	*DOCK8*	AR	T cell lymphopenia, reduced Th17 cells, increased total B cells, reduced switched memory B cells	Elevated IgE, low IgM	Severe eczema, recurrent respiratory infections, cutaneous viral, fungal, and staphylococcal infections, severe allergy	Increased risk for malignancies
PGM3 deficiency	*PGM3*	AR	T cell lymphopenia, low B and memory B cells	Elevated IgE, normal/elevated IgG and IgA	Severe eczema, recurrent skin and pulmonary infections, severe allergy	Facial features (wide nostrils and prominent lips), skeletal abnormalities(scoliosis), neurocognitive impairment (development delay, low intelligence quotient)
CARD11 deficiency	*DOCK8*	AD LOF(DN)	T cell lymphopenia, Th2 skewed immune response, normal or low B cells	Elevated IgE, normal/low IgG	Severe eczema, skin viral infections, recurrent pulmonary infections, severe allergy	lymphoma

Notes: IgE: Immunoglobulin E; AD: autosomal dominant; LOF: loss-of-function; DN: dominant negative; AR: autosomal recessive.

## Abbreviation

PIDDs	Primary immunodeficiency diseases
HIES	Hyper- IgE syndromes
IgE	Immunoglobulin E
WAS	Wiskott-Aldrich syndrome
IPEX	Immune dysregulation, polyendocrinopathy, enteropathy, X-linked
OS	Omenn syndrome
XLA	X-linked agammaglobulinemia
AD	Autosomal dominant
AR	Autosomal recessive
STAT	Signal transducer and activator of transcription-3
DN	Dominant negative
PGM3	Phosphoglucomutase 3
DOCK8	Dedicator of cytokinesis-8
IUIS	International Union of Immunological Societies
SAD	Severe atopic dermatitis
LOF	Homozygous loss-of-function
ZNF	Zinc finger protein
TGF-β	Transforming growth factor β
Tregs	Regulatory T-cells
ERBB2IP	ERBB2-interacting protei
CID	Combined immunodeficiency
HPV	Human papillomavirus
HSV	Human simplex viruses
CARD11	Caspase recruitment domain family member 11
CMC	Chronic mucocutaneous candidiasis
HSCT	Hematopoietic stem cell transplantation
WASp	Wiskott-Aldrich syndrome protein
HGMD	Human Gene Mutation Database
XLT	X-linked thrombocytopenia
GT	Gene therapy
GvHD	Graft-versus-host disease
FOXP3	Forkhead box P3
GH	Growth hormone
GHI	Growth hormone insensitivity
DGS	DiGeorge syndrome
BTK	Bruton tyrosine kinase
IVIG	Intravenous immunoglobulin
SCIG	subcutaneous immunoglobulin
AE	Acrodermatitis enteropathy
HLCSD	Holocarboxylase synthetase deficiency
BTD	Biotinidase deficiency
PD	Prolidase deficiency
SAM syndrome	Severe dermatitis multiple allergies and metabolic wasting syndrome
NS	Netherton syndrome
SPINK5	Serine protease inhibitor of Kazal type 5
TSLP	Thymic stromal lymphopoietin
ILC	Icthyosis linearis circumflexa
PSS-B	Peeling skin syndrome type B
CDSN	Corneodesmosin
